# High Exogenous Antioxidant, Restorative Treatment (Heart) for Prevention of the Six Stages of Heart Failure: The Heart Diet

**DOI:** 10.3390/antiox11081464

**Published:** 2022-07-27

**Authors:** Ram B. Singh, Jan Fedacko, Dominik Pella, Ghizal Fatima, Galal Elkilany, Mahmood Moshiri, Krasimira Hristova, Patrik Jakabcin, Natalia Vaňova

**Affiliations:** 1 Halberg Hospital and Research Institute, Moradabad 244001, India; rbs@tsimtsoum.net; 2 Department of Gerontology and Geriatric, Medipark, University Research Park, PJ Safarik University, 040-11 Kosice, Slovakia; 3 Department of Cardiology, Faculty of Medicine and East Slovak, Institute for Cardiovascular Disease, PJ Safarik University, 040-11 Kosice, Slovakia; dominik.pella@gmail.com; 4 Department of Biotechnology, Era University, Lucknow 226001, India; ghizalfatima8@gmail.com; 5 International College of Cardiology, Laplace, LA 90001, USA; galal.elkilany@gmail.com; 6 International College of Cardiology, Richmond Hill, ON LL-9955, Canada; moshiri@nextpharmainc.com; 7 Department of Cardiology, National University Hospital, 1000 Sofia, Bulgaria; khristovabg@yahoo.com; 8 Department of Social and Clinical Pharmacy, Faculty of Pharmacy in Hradec Králové, Charles University, 10000 Prague, Czech Republic; jakabcin.patrik@gmail.com; 9 Department of Internal Medicine UPJS MF and AGEL Hospital, Research Park, PJ Safaric University, 040-11 Kosice, Slovakia; nvanova@nke.agel.sk

**Keywords:** Western diet, cardiomyocyte, oxidative stress, bioactive agents, dietary fat, heart hypertrophy, cardiac failure

## Abstract

The exact pathophysiology of heart failure (HF) is not yet known. Western diet, characterized by highly sweetened foods, as well as being rich in fat, fried foods, red meat and processed meat, eggs, and sweet beverages, may cause inflammation, leading to oxidative dysfunction in the cardiac ultra-structure. Oxidative function of the myocardium and how oxidative dysfunction causes physio-pathological remodeling, leading to HF, is not well known. Antioxidants, such as polyphenolics and flavonoids, omega-3 fatty acids, and other micronutrients that are rich in Indo-Mediterranean-type diets, could be protective in sustaining the oxidative functions of the heart. The cardiomyocytes use glucose and fatty acids for the physiological functions depending upon the metabolic requirements of the heart. Apart from toxicity due to glucose, lipotoxicity also adversely affects the cardiomyocytes, which worsen in the presence of deficiency of endogenous antioxidants and deficiency of exogenous antioxidant nutrients in the diet. The high-sugar-and-high-fat-induced production of ceramide, advanced glycation end products (AGE) and triamino-methyl-N-oxide (TMAO) can predispose individuals to oxidative dysfunction and Ca-overloading. The alteration in the biology may start with normal cardiac cell remodeling to biological remodeling due to inflammation. An increase in the fat content of a diet in combination with inducible nitric oxide synthase (NOSi) via N-arginine methyl ester has been found to preserve the ejection fraction in HF. It is proposed that a greater intake of high exogenous antioxidant restorative treatment (HEART) diet, polyphenolics and flavonoids, as well as cessation of red meat intake and egg, can cause improvement in the oxidative function of the heart, by inhibiting oxidative damage to lipids, proteins and DNA in the cell, resulting in beneficial effects in the early stage of the Six Stages of HF. There is an unmet need to conduct cohort studies and randomized, controlled studies to demonstrate the role of the HEART diet in the treatment of HF.

In the ancient scripture Bhagavad Gita (3000 BCE) in India, Sattvic diets were advised for health, happiness, peace and longevity (https://www.holy-bhagavad-gita.org/chapter/17/verse/8, accessed on 15 June 2022).

## 1. In Sanskrit

Aayuh satvabalarogyam, sukhpreetiviverchanah,

Rasyah snigdhah sthirah hradyah aharah satvikpriyah [1,2,3].

## 2. Translation in English

Sattvic foods are full of nutrients, they are very juicy, good in taste and increase the longevity, wisdom, power, health, happiness, peace and love.

The Sattvic dietary patterns are characterized by plenty of fresh, nutrient-rich foods, including vegetables, fruits, sprouted grains, almonds, fresh fruit juices, seeds, legumes, curd, honey, Panchamrat (mix of milk and curd, with honey, clarified butter and basil leaves) and tea made from herbs. These foods present in the Sattvic diet are well known to impact the oxidative function of the body, which involves the heart.

Oxidative stress and oxidative function are considered important in the physiopathology and progression of heart failure (HF) [1]. Oxidative stress is characterized by the imbalance, in the generation of reactive oxygen species (ROS), and the availability of the endogenous antioxidant enzymes and other defences. Physiologically, a small amount of ROS is generated in the cells, for cell signaling, that is spontaneously decreased by the endogenous antioxidant defences. However, in disease states, the generation of ROS is greater than the buffering capability of the endogenous antioxidant defences, causing cell damage, protein and peroxidation of lipids, damage to DNA with irreversible cell damage and cell apoptosis, indicating loss in cardiac cells [1]. This physiopathology can be seen in the heart, showing an increase in high-sensitive (hs) troponin during the progression of HF [1,4]. In previous research, it has been possible to target oxidative stress for improvement in outcome in HF patients, with marginal success. The exact cause of the failure of these studies, in determining any protective effects, is not clear. It seems that it is plausible to explain that currently used methods, which inhibit oxidative stress by exogenous means against the overproduction of ROS, or supplementation of exogenous antioxidants, are not optimal enough. It seems that enhancing the endogenous capacity of antioxidants could be an additional approach for treatment and prevention [1,4]. This communication provides a critical review of oxidative stress and oxidative function in the HF pathophysiology and the strategies used until now for targeting this pathway. Interestingly, accurate modulation of endogenous antioxidants, possibly via high exogenous, antioxidant restorative treatment (HEART) diet, may lead to novel therapeutic strategies for possible improved outcome in patients with HF.

## 3. Oxidative Dysfunction in Heart Failure

It seems that behavioral risk factors such as Western diet, tobacco and alcohol intake, short sleep, and mental stress can cause an overproduction of free radicals, oxidative myocardial dysfunction and inflammation, which may alter the twist of the heart due to cardiomyocyte dysfunction and physiological remodeling initially [5]. The intracellular oxidative homeostasis in the cardiac cells is closely regulated by the production of ROS with limited intracellular defense mechanisms.

If the oxidative dysfunction continues, it may lead to pathological remodeling with cardiac damage in the form of increased high-sensitivity (hs) troponin T, in cardiac cells causing abnormalities in the global longitudinal strain [6]. In the cardiac cells, an overproduction of ROS may lead to the development and progression of maladaptive myocardial remodeling, which may be an early stage of HF [1,4]. Oxidative stress and ROS directly cause inflammation and impair the electrophysiology of the heart by targeting contractile machinery and cardiac components via the dysfunction of proteins that are crucial to excitation–contraction coupling, including sodium channels, L-type calcium channels, potassium channels, and the sodium–calcium exchanges [1,4,5,6,7]. Oxidative stress may also cause alteration in the activity of the sarcoplasmic reticulum Ca^2+^-adenosine triphosphatase (SERCA) as well as reduce myofilament calcium sensitivity [7]. In addition, oxidative stress can induce an energy deficit by influencing the protein function related to metabolism of energy [7]. Oxidative dysfunction may facilitate a pro-fibrotic function, as adaptation, by causing the proliferation of fibroblasts in the heart and matrix metallo-proteinases for extracellular remodeling, which may be the beginning of the hypertrophy of the heart [1,4].

**Figure 1:**
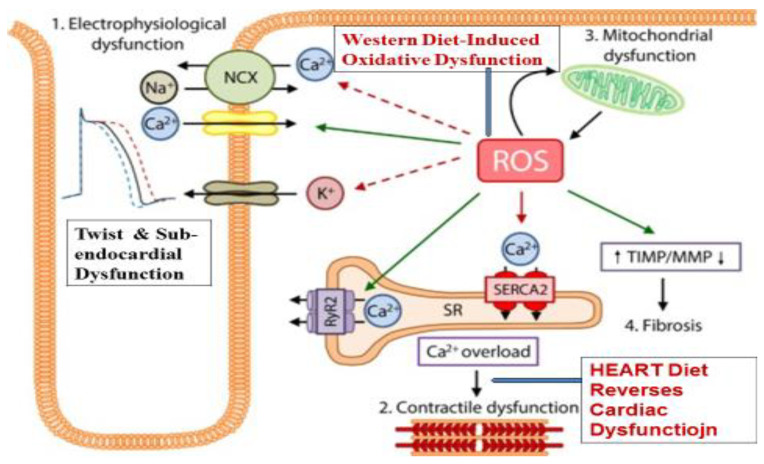
Oxidative dysfunction in the heart due to Western diet, with decrease in antioxidant defences, causing mitochondrial dysfunction, leading to electrophysiological dysfunction with twist and sub-endocardialdysfunction. Exogenous antioxidants (HEART diet) improve antioxidative function with reduced Ca overloading and reversal of mitochondrial and electrophysiological dysfunction. (Modified from Reference [1], VanderPolA, Euro J Heart Failure 2019, under the license http://creativecommons.org/licenses/by-nc/4.0/, accessed on 15 June 2022).

It seems that the production of ROS in the heart is primarily completed by the mitochondria, xanthine oxidase, NADPH oxidases, and uncoupled nitric oxide synthase (NOS) [1]. The electron transport chain of the mitochondria may cause an overproduction of superoxide anion, contributing to cardiomyocyte damage with an increase in myocardial injury after an acute myocardial infarction [1]. There may be an increase in oxidative stress with an increased expression and activity of NADPH oxidase, due to multiple environmental and biological factors, such as angiotensin II, endothelin-1, mechanical stretch and tumor necrosis factor (TNF)-α [1,4,5,6,7]. The expression of xanthine oxidase and its activity is also increased due to damaging effects of behavioral risk factors such as tobacco intake and alcoholism in the heart exposed to these risk factors. It is proposed that oxidative dysfunction with increased oxidative stress may be the first stage of HF, which may be associated with cardiac damage and dysfunctional twist [5,6,7,8]. If there is a lower availability of endogenous antioxidants, super-oxide-dismutase (SOD), glutathione-peroxidase (GPS) and catalase or coenzyme Q10, it may cause the worsening of cardiac function, resulting in sub-endocardial damage, which may be the second stage of HF [8,9], There may be an uncoupling of the NOS with structural instability, which further increases the generation of ROS, leading to left ventricular (LV) enlargement, dysfunction in the contraction [1], and remodeling of LV [1,4]. If the cardiac damage continues, it may lead to increased sympathetic activity with decline in parasympathetic activity causing neuro-hormonal dysfunction [1,4,5,6,7,8]. Interestingly, the protective factors, such as the HEART diet may prevent the development of HF, if administered in one or the other of the early stages of Six Stages of HF given in Table 1 [2,9,10,11]. This mechanism is more clearly evident in Figure 1.

## 4. Left Ventricular Twist as Function of the Heart

Richard Lower FRCP (1631–1691) was the first to publish the twisting motion of the LV, in 1669, as “the wringing of a linen cloth to squeeze out the water”, which continues to intrigue the experts in their quest to understand cardiac function [13,14,15]. Apart from speckle tracking echocardiography (STE), magnetic resonance imaging (MRI) may be used to examine LV twist [15,16]. It appears to be crucial to examine twist function to understand the oxidative function of the heart, which would require quantification of the LV twist. The cardiac twist or torsion represents the mean longitudinal gradient of the net difference in the clockwise and counterclockwise rotation of the apex and base of the LV, as viewed from the apex of the left ventricle. The LV twist deforms the sub-endocardial fiber matrix, resulting in the storage of potential energy. A further deformation in the recoil of twist may cause the release of restoring forces, which contributes to diastolic relaxation of the LV with early diastolic filling [16]. Interestingly, systolic function may not be entirely normal, despite the normal ejection fraction (EF). There may be a decline in the left ventricular systolic long-axis at earlier stages, followed by evidence of more greater, subtle defects. On physical training, with decreased augmentation of function in the long-axis, impairment in systolic twist, decreased global strain, and electromechanical dys-synchrony will reduce the myocardial systolic reserve [16,17,18]. The twist function may alter during oxidative myocardial dysfunction, which may be an early marker of HF.

The physiology of twist mechanics indicate that LV twists in systole store optimal energy and, during the recoils (untwists) in diastole, cause energy release [17]. It seems that left ventricular ejection is aided by twist and untwist, which is helpful for the relaxation and filling of the ventricle. Thus, twist or torsion and rotation are crucial in cardiac contraction mechanics. Torsion or twist is accompanied by the wringing motion of the heart in its long axis produced by contraction of the myofibers in the wall of LV [13]. The apex and the base of the heart, during initial isovolumic contraction, both rotate in a counterclockwise method, if observed from apex to base. However, in the normal heart, the base of the heart has clockwise rotation during systole and the apex of the heart has counterclockwise rotation, causing a wringing movement. The cardiologists are not able to understand the utility of the twist function in clinical practice, which may be due to the problems in the measurement of cardiac rotation and torsion in the clinic [3,18]. It seems that three-dimensional STE may be an alternative method to assess the twist function, during plane motion. However, it seems that the measurement of the twist function would enhance our knowledge of physiological mechanics of the heart, such as the early diagnosis of abnormality in the rotation, indicating sub-endocardial dysfunction, the second stage of the six stages of HF, that may occur due to behavioral risk factors such as tobacco and Western diet. These risk factors may be also helpful in exploring the secrets of the diastole (a Rosetta stone), which could be a new concept in diastolic function and diastolic HF, via STE, in the light of neuro-humoral dysfunction [3,12,18,19,20]. It seems that the physician needs to have a closer look to understand the physio-pathogenesis of oxidative myocardial function and cardiac dysfunction, in particular, the LV twist and decline in myocardial strain [8]. There is an unmet need to use rotation and twist, as well as reversible sub-endocardial and diastole dysfunction in the diastole, via STE, as new markers of cardiac function, in the presence of oxidative dysfunction of the myocardium [12,19,20]. These six stages of oxidative myocardial dysfunction, proposed by us, may be used to begin therapy, possibly for the primordial prevention of HF, Table 1.

## 5. Oxidative Dysfunction and Inflammation as Targets for Therapeutic Antioxidants

**Figure 2:**
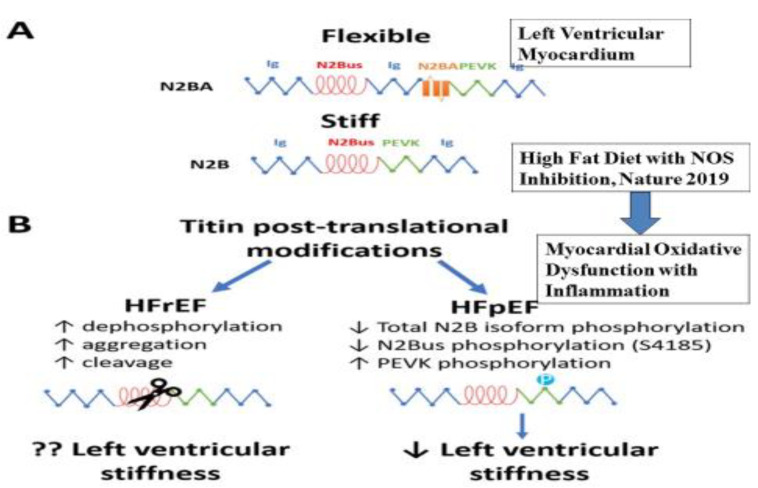
Myocardial oxidative dysfunction due to high-fat diet causing nitric oxide synthase (NOS) inhibition leading to heart failure with preserved ejection fraction (HFpEF). (**A**) Alternative isoforms of titin in Left Ventricular Myocardium. (**B**) Post-translational modifications of Titin and their effect on left HFrEF and HFpEF (Adapted from Reference [23] Simmonds, S.J., et al., Cells. 2020; 9: 242. 10.3390/cells9010242, under liscence http://creativecommons.org/licenses/by/4.0/, accessed on 15 June 2022).

Preclinical and clinical studies indicate that several therapeutic options are available to treat oxidative stress-associated cardiovascular diseases (CVDs) [1,4,5]. Many of the antioxidants, such as dietary content of phytochemicals, and novel polyphenols, have been examined for therapy, in view of the risk factors and inflammatory mediators of HF [4,21,22]. Apart from these, new therapeutic methods such as miRNA and nano-medicine are also available for the treatment of CVDs, in particular, HF, which may be tried, during the early stages of the Six Stages of HF (Table 1). It seems that an increase in free fatty acids and oxidative dysfunction with reference to variability in biomarkers such as glucose levels, and levels of oxidative stress, predispose individuals to multifold greater inflammation and immune deficiency, leading to cardiac cell apoptosis and heart failure (HF) [23,24,25]. Decline in immunological responses may result in damage to other body systems contributing in diseases of associated body systems [23,24,25]. Free radicals are known to damage the cell membranes, causing the development of intracellular Ca^2+^ overload, activation of proteases and phospholipases, and alterations in mitochondrial gene expression in the cardiac cells, predisposing individuals to cardiomyocyte dysfunction [23,24,25,26,27]. Deficiency of protective antioxidants may predispose individuals to oxidative damage to proteins, enzymes, fatty acids and DNA [28,29,30]. It is possible that the cell damage may be reversed by the HEART diet. Experimental and epidemiological studies have also demonstrated that Western-type diets characterized by high sugar and refined carbohydrates with a high glycemic index, as well as high-fat diet, red meat and preserved meat, may predispose individuals to increased risk of HF [28,29,30,31,32,33,34,35,36], Figure 2.

Apart from endogenous antioxidant defences, several exogenous antioxidants are available that may be administered for the treatment of HF. Since therapy with individual antioxidants in patients with CVDs has only had limited success, there is a need to determine the role of the Mediterranean diet, such as the HEART diet, in the management of HF, Table 2.

## 6. Effects of HEART Diet in Heart Failure

The mechanisms responsible for the beneficial effects of antioxidants or the HEART diet in HF may be a decline in oxidative stress and cardiac inflammation, a reduction in mitochondrial dysfunction, improved Ca^2+^ homeostasis, increased survival signaling, and an increase in sirtuin 1 activity [1]. It seems that all these mechanisms are heightened in conjunction with excessive oxidative stress due to the intake of a Western type of diet derived primarily by overexpression of nicotinamide adenine dinucleotide phosphate (NADPH)-oxidases (Nox) and an increase in mitochondrial-derived ROS that are major drivers of HF [1,4]. It is possible that the HEART diet reverses the detrimental effects of oxidative stress, while cellular antioxidants such as vitamin E, C and CoQ10 and detoxifying enzymes neutralize ROS and ameliorate cytotoxic conditions [1,4]. These enzymes include superoxide dismutase (SOD), catalase, glutathione S-transferase, glutathione peroxidase (GPx), heme oxygenase (HO)-1 and NADPH dehydrogenase quinone 1 (NQO1), which are mostly co-regulated by Sirt1 and nuclear factor erythroid 2-related factor 2 (Nrf2) [1,4]. Since there is a state of exacerbated oxidative stress and inflammation in HF, the detoxifying system is overwhelmed, as NF-κB overexpression can inhibit Nrf2 nuclear activity, and vice-versa [4]. Inflammation facilitates macrophage recruitment into the myocardium via chemoattractants and also leads to the differentiation of fibroblasts into myofibroblasts, promoting fibrosis [4]. Collectively, these signaling effects lead to cardiomyocyte oxidative dysfunction, cardiac hypertrophy, apoptosis, pro-fibrotic signaling and, at the organ level, reduced functional capacity. It seems that mediating the inflammatory and antioxidant responses and other mechanisms via the HEART diet is of major therapeutic relevance in HF.

There are multiple pathways by which nutritional factors can have adverse or beneficial effects in the development of CVDs [24,25,26,27,28]. It seems that beyond drug therapy, the nutritional status of the patients of HF can also influence the effects of therapy due to cardioprotective factors such as coenzyme Q10 and resveratrol, nutrients in the cardiac tissues [24,25,26]. Apart from these nutrients, certain factors in the brain, such as the renin–angiotensin–aldosterone system (RAAS), can act as an oxidant, leading to an increase in inflammation in the neurons [27,28]. Inflammation in the brain as part of neuro-hormonal dysfunction may activate the prefrontal cortex and amygdala, leading to an increase in brain neuropeptide, angiotensinogen II (ANG II). These pro-inflammatory factors can damage the hippocampus, pre-sympathetic neurons in the paraventricular nucleus as well as preganglionic sympathetic neurons. Since the Mediterranean diet is known to protect brain function by its benefits in depression and dementia, it poses the possibility that the HEART diet, which is an improved Mediterranean-style diet, may provide greater beneficial effect on brain-related mechanisms of HF [29,30]. There is existing evidence that diets deficient in omega-3 fatty acids [31] and whole grains [32], as well diets with an excess of red meat [33], processed meat [34], and high-glycemic-index foods [35], can predispose individuals to HF.

## 7. Dietary Fat and Risk of Heart Failure

Recent and previous experiments published in *Nature* confirm the role of nutrition in the pathogenesis of CVDs and diabetes as well as in HF [37,38]. Experimental studies confirm that high dietary total and saturated fat and high glucose intake have an adverse effect on cardiac cell function, and high fat intake along with arginine may be associated with increased risk of HFpEF, in an attempt to prevent a decline in the ejection fraction, as a mechanism of molecular adaptation [38]. Such diets may begin their adverse effects by causing twist dysfunction (Stage 1 HF) and later on sub-endocardial dysfunction (Stage 2 of HF) due to oxidative damage of proteins, enzymes, fatty acids and DNA. In a previous experimental study, a high-fat diet given to fathers in mice showed adverse effects on offspring [37]. The findings revealed that a chronic high-fat diet administered to fathers programs β-cell dysfunction in female rat offspring and induces obesity-impaired glucose tolerance [IGT], insulin resistance that worsened with time, relative to controls. Administration of this diet was associated with alteration in the expression of 642 pancreatic islet genes in the offspring of female adults. These genes were related to 13 functional clusters, such as ATP binding, cation, intracellular transport and cytoskeeton [37]. Further analysis of 2492 genes with variable expression, showed the participation of pathways related to Ca-MAPK and MnT signaling, apoptosis and the cell cycle. It has also been observed that the gut flora metabolism of phosphatidylcholine promotes CVDs, including HF [39].

HF with preserved ejection fraction (HFpEF) is difficult to treat and its exact pathogenesis is unknown. In the pathophysiology of HFpEF, fibrosis and the rigidification of titin are two important factors that predispose individuals to high diastolic left ventricular stiffness, which may preserve the ejection fraction as a mechanism of adaptation [40,41,42,43,44,45,46]. A recent study of a mouse model indicated an alternative path, with implications for new experimental strategies [40]. This experiment showed that metabolic stress added with hypertensive stress, produced due to the effect of increased fat diet and nitric oxide (NO) synthase inhibition by N^[w]^-nitro-l-arginine methyl ester (L-NAME) may alter the pathophysiology of HF. The mouse model simulates the numerous systemic and cardiovascular features of human HFpEF [31]. Interestingly, one of the response effectors due to unfolded protein, the spliced form of X-box binding protein 1 (Xbp1s), may decrease in the heart of both experimental and human HFpEF. Treatment with drug or suppression of the gene of iNOS, or the cardiac cell-restricted manifestation of Xbp1s, was able to inhibit the production of the HFpEF phenotype, unveiling iNOS-induced dysfunction of IRE1α-Xbp1s as an important mechanism of dysfunction of cardiac cells in HFpEF [47]. It seems that HFpEF may be a nutritional adaptation of the cardiac cells by which they preserve the myocardial function without any reduction in the ejection fraction. It is possible that increased supplementation of monounsaturated fatty acids and w-3 fatty acids with flavonoids, resveratrol and no red meat and egg (betaine and choline) might cause an additional benefit in the cardiac cell function, with improved twist function and sub-endocardial function, resulting in the inhibition of HFrEF [31,37,38,39,47,48,49,50]. The role of choline in the pathogenesis of HF is discussed below.

In all CVDs and diabetes metabolic processes, diet holds promise for the discovery of new pathways that link the primary risk factors to disease processes [37,38,39,48]. There is evidence that metabolites of the dietary lipid phosphatidylcholine, betaine, choline and trimethylamine *N*-oxide (TMAO) may have a major role in the pathophysiology of CVDs [39,48]. Dietary supplementation of mice with choline, TMAO or betaine predisposed individuals to the upregulation of several macrophage scavenger receptors linked to the pathophysiology of atherosclerosis [39]. However, administration of TMAO or phosphatidylcholine increased the process of atherosclerosis. Experimental studies in germ-free mice showed a critical role for dietary choline and gut flora in TMAO production, which augmented cholesterol accumulation in the macrophage cholesterol, leading to foam cell formation. In the atherosclerosis-prone mice experiment, suppression of intestinal microflora was associated with inhibition of dietary-choline-enhanced atherosclerosis. There are variations in the gene-controlled expression of flavin monooxygenases, an enzymatic source of TMAO, segregated with atherosclerosis in mice with hyperlipidemia [39]. This discovery indicates a relation of gut-flora-dependent metabolic function of phosphatidylcholine in the foods and pathophysiology of CVDs. It is possible that new biomarkers may be developed for making an early diagnosis of CVDs and diabetes, which may be useful in developing new therapeutic approaches for the prevention of HF. TMAO is produced in the body, in a co-metabolic pathway, concerned with microbial-mammalian, from the digestion of foods with meats having dietary quaternary amines, such as betaine, phosphatidylcholine and L-carnitine [46]. Intake of fish has been found to be protective against CVDs and diabetes but it provides a direct significant source of TMAO. It is possible that adverse effects of TAMO, such as oxidative stress and inflammation, are neutralized due to the presence of omega-3 fatty acids and peptides in the fish, leading to overall benefits. There may be discrepancies and inconsistencies in the recent investigations, and the role of TMAO has been questioned in some diseases, because its precursor L-carnitine has been found to be beneficial in CVDs [46]. Recent experimental and epidemiological studies on the effects of TMAO indicate that it may have beneficial effects in the presence of a diet, which is protective for the microbiome [46]. In obesity, the relative proportion of Bacteroidetes is decreased compared to lean subjects, and that this proportion increases with loss of weight on two types of low-energy diet [51]. It is possible that obesity has a microbial part, which might have important therapeutic potentials [51].

**Figure 3:**
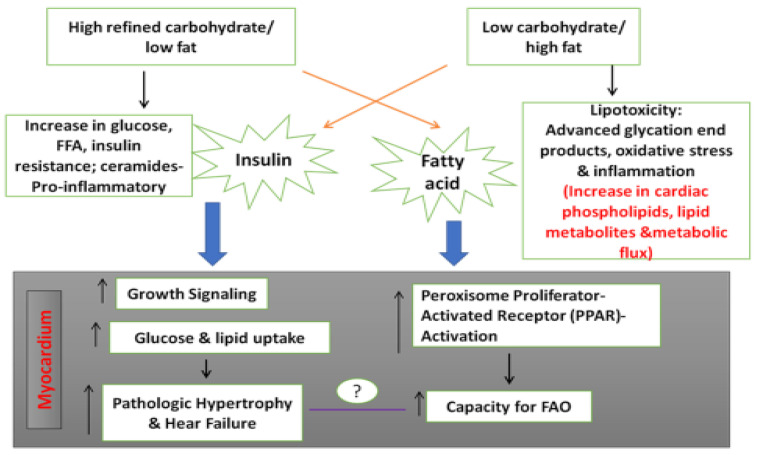
Effects of high-glucose or high-saturated-fat diets on development of cardiomyocyte and cardiac hypertrophy. (Modified from Reference [48], Stanley et al., Circ Res 2012, *American Heart Association*, Journal).

The effects of high-glucose, low-fat or high-saturated-fat-and-low-carbohydrate diets may cause oxidative dysfunction in the cardiac cells due to augmentation of protein synthesis and a decrease in protein breakdown. These alterations may predispose individuals to twist dysfunction, sub-endocardial dysfunction, resulting in cardiomyocyte remodeling, leading to cardiac hypertrophy and HF [48] (Figure 3).

Western-diet-induced inflammation of the heart may mediate the activation of multiple mechanisms that predispose individuals to CVDs, including CHF [37,38,39,47,48,49]. Apart from glucotoxicity, lipotoxicity may be associated with the activation of the receptor for advanced glycation end products (RAGE) due to an increase in advanced glycation end products (AGEs) predisposing individuals to oxidative stress and inflammation [41]. In earlier studies, such actions have largely been ascribed to fat deposition due to Western diet and the accumulation of AGEs. Subsequently, there is RAGE activation, which can represent necessary mediators of cardiac cell injury leading to the hypertrophy of cardiac cells. An experimental study included *RAGE* knockout mice that were administered either a 7% fat standard diet or a high-fat Western diet containing 21% fat [41]. Animals who had a high-fat diet were again randomized to get the AGE inhibitor alagebrium chloride (1 mg·kg^−1^·day^−1^), and the follow-up period was 16 wks. The group given a Western high-fat diet was associated with hypertrophy of heart, mitochondrial-dependent superoxide production, inflammation and accumulation of cardiac AGE. The mice (*RAGE*-KO) given a Western diet also gained body weight, turning obese with the accumulation of lipids in the myocardium and hypertrophy of cardiomyocyte, with oxidative stress and inflammation, which were reduced when compared with wild-type mice. Interestingly, both strains of mice receiving alagebrium chloride revealed lower oxidative stress and inflammation and showed a decline in cardiac advanced glycation end-products and receptors of advanced glycation end-products. It is clear that advanced glycation end-products may show necessary mediators of injury of the heart while receiving a fast-food-containing Western diet [41]. These studies indicate a potential utility of methods for decreasing AGE that are protective against cardiac disease.

In view of the increasing evidence, indicating that dietary fatty acid intake influences the development and progression of HF, there is an unmet need to determine which fat is lipotoxic and which one has lipo-protective effects [40]. Experimental research indicates that those animals without obesity, substituting refined, high sugar and fat, may decrease or inhibit an increase of LV and dysfunction in contraction as a result of high blood pressure, myocardial infarction or cardiomyopathy [40]. However, adding n-3 fatty acids from fish causes alteration in the composition of cell membrane phospholipid fatty acid, improves mitochondrial function, diminishes the development of new HF, and inhibits the progression of established HF [31,52]. There is evidence that high consumption of n-3 fatty acids can be effective in the treatment HF. Omega-3 fatty acids in the diet can alter the composition of cardiac mitochondrial phospholipid and cause a delay in the Ca^2+^-induced transition of cell membrane permeability [52]. It is proposed that increased availability of magnesium (Mg^2+^) may activate Ca-Mg-ATPASE and improve anti-inflammatory effects of these fatty acids. In an experiment, treatment of a normal rat with DHA + EPA (70:30 ratio; 2.3% of energy intake) for 36 weeks, delayed Ca^2+^-induced mitochondrial permeability transition pore (MPTP) opening in isolated cardiac mitochondria [43]. It is observed as an increase in the capacity for mitochondrial Ca^2+^ uptake.

**Figure 4:**
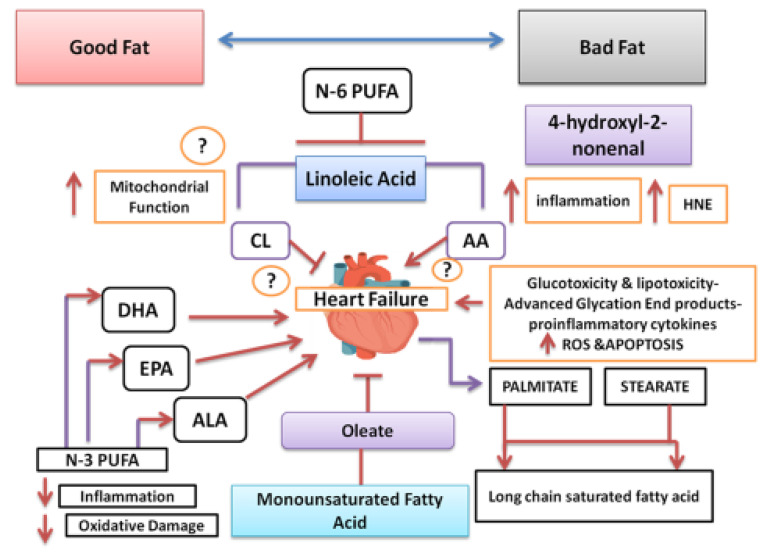
Depiction of mechanisms of how a high-saturated-fat and high-glucose diet is necessary for causing progression of heart failure, and good fat with flavonoids causes improvement. (Modified from Stanley et al., Circ Res, 2012, Reference [48], American Heart Association.) TMAO = Trimethylamino-N-Oxide.

It seems that greater consumption of oleic acid or n-6 fat has also been found to provide improvement in cardiac hypertrophy [40]. This finding may indicate a decrease in oxidative stress and inflammation and better resistance to transition in mitochondrial permeability. It seems that the related mechanisms are complex, which could be on account of adaptation of the heart, in a situation, on saturated fat diets. There is an unmet need to have complete understanding of the effects of different types of dietary fats on phospholipids in the cell membrane of cardiac cells, metabolites of lipids and metabolic functions in the failing as well as in the normal heart. It is likely that existing nutrients such as w-3 fatty acids, flavonoids, and coenzyme Q10 may inhibit the lipotoxicity caused by saturated fat and delay the progression of cardiac hypertrophy, by improving twist function and sub-endocardial function, which may cause HFpEF, in place of HFrEF. It seems that changes in fat consumption could be crucial in the management of HF. The influence of the HEART diet or Western type of diet on cardiac cells could be dependent on pro-inflammatory biomarkers that can damage cardiomyocytes. High-glucose or fast-food diets induce an increase in ceramides and high levels of TMAO on account of greater consumption of red meat (L Carnitine) and egg (phosphatidylcholine) as well as a rise in AGE products, caused by increased saturated fat in the diets that are new biomarkers of dilatation of the heart [40,43,44,45,46]. These oxidants should be duly inhibited by new therapies for the prevention of cardiac hypertrophy, which may also produce HFpEF. Long-term follow-up studies and preclinical studies are required to determine the role of the HEART type of diets and unhealthy diets, with reference to fatty acids content for this group of vulnerable population (Figure 4).

## 8. Mechanisms of Diet and Obesity in Heart Failure

There is evidence that the Western type of diet is a risk factor of obesity, whereas Mediterranean-style diets may have protective effects on obesity and HF [46,52,53]. It is possible to create diet-induced obesity in animal experiments, in selected strains, by use of a diet that is rich in fat (about 40% to 50% of energy, verses 10–15%) in conjunction with sugar (~20% to 30% sucrose). It appears that obesity may have complex influences on the cardiac ultra-structure, mediated via alterations in circulating hormones, impairment in arterial muscle function and changes in the autonomic regulation of the heart and vessels [51]. Therefore, experimental research that determines the effects of high fat/low carbohydrate intake should be examined with caution, because obesity induced by diet may have confounders [51]. If the obesity is absent, then substituting carbohydrate with fat in the diet may inhibit or decrease the progression of HF. This benefit occurs by preserving twist function and/or sub-endocardial function, which develops in response to hypertension or myocardial infarction, indicating that sugar may have more adverse effects than saturated fat. Thus, feeding a high-fat/low-carbohydrate diet to normal healthy rats and mice generally has no adverse effects on the heart if there is not concomitant obesity. In some of the experiments with obesity, produced via high-fat feeding, there are often no pathological changes on the heart [51], or there may be mild LVH and dysfunction in the contraction with hypertension and increase in leptin [51,54,55]. Experiments in transgenic mice and leptin-deficient Zucker fatty rats demonstrated that if fatty acid uptake in the myocardium and/or esterification is increased to supra-physiological concentrations, it may cause accumulation of triglycerides and lipid intermediates in the cells, leading to dysfunction in the contraction of the heart. There may be hypertrophy of cardiomyocytes, apoptosis, and HF [56,57]. There is only limited evidence in humans on the clinical value of these findings in relation to fatty acids in the diet and the progression of HF. The mechanisms of lipotoxicity in the animal models with altered genes do not appear to be relevant to physiopathology, in the normal or failing heart in humans [56,57,58].

## 9. Epidemiological Studies on Diet and Risk of Heart Failure

There are limited known large-scale epidemiological studies indicating the role of dietary factors in the pathogenesis of HF [46,59,60,61]. The dietary quality of persons with HF was examined in the NHANES 1999–2006; among the 574 patients, the mean age was 70 years, with 52% being women [46]. The intake of mean sodium was 2719 mg, with 34% consuming less than 2000 mg per day. The intake of potassium was a mean of 2367 mg/day, without consideration for the type of diuretic used or renal disease status. The intake of other nutrients, as per the guidelines, was low for some nutrients—13% for calcium, 10% for magnesium, 2% for fish oils, and 4% for fiber—but high (13%) for saturated fat [27,46]. The dietary quality of persons with self-reported HF was poor. In a case control study from USA among 246 patients, aged mean 61.5 years, with 67% in New York Heart Association class III/IV HF, micronutrient deficiencies were determined [59]. Among 246 patients, 29.8% had hospitalization or death at one year follow-up, which included 44.3% in the subgroup with high-deficiency and 25.1% in the rest of them. The distribution of survival revealed significant differences (log rank, *p* = 0.0065). It is possible to conclude from this study that the quality of dietary intakes of the patients with HF may be crucial in determining outcomes [59]. In another study, comprising 118 patients, 54% were males, aged 66 years (median), with a median ejection fraction of 45% (30–60%), and 49% of patients had CAD [62]. There was a significant association for PUFA; adjusted hazard ratio (HR), 0.67, for consumption as SFA; adjusted HR, 1.15, for consumption as percentage of daily energy. The median of consumption of daily energy was 8.2% for SFA and 5.3% for PUFAs. Interestingly, the consumption of SFA and PUFAs was positively co-related with 1-year all-cause mortality in CHF patients [62]. It is possible that decreasing dietary saturated fat with an increase in PUFA consumption should be the strategy in these subjects.

Recently, Hristova et al. re-emphasized the role of nutritional modulators among patients with CHF, because these patients may suffer from weight loss as well as cachexia, which is associated with deficiency of antioxidant vitamins, such as magnesium, potassium, and vitamin D, as well as fiber and flavonoids, apart from general malnutrition. Hristova et al., as well as Fedacko et al., reported the risk factors and inflammatory mediators of HF among 116 patients from India, in which only little attention was paid to nutritional risk factors in HF [21,22]. Although dietary intakes were not reported in this paper, personal communication revealed that these patients were consuming a significantly lower quantity of vegetables, fruits, nuts and legumes (<400 g/day) [22]. However, beyond these factors, several studies have demonstrated that following an injury to the cardiomyocyte during a disease, an intense inflammatory response occurs that predisposes individuals to further damage and the progression of cardiac dilatation and dysfunction [21,22]. The cell debris, such as extracellular ATP, released during tissue injury induces conformational changes in the components of the inflammation in cardiac tissue, which may worsen if there is deficiency of antioxidant nutrients in the tissue [21,22]. The harmful biomarkers in failing cardiac cells are as follows: cryopyrin (NLRP3 encodes cryopyrin, which belongs to an emerging family of danger sensors, called NLRs = NOD-like receptors, which are sensor proteins) and the apoptosis-associated speck-like protein containing a CARD (C-terminal caspase-recruitment domain) (ASC), adaptor proteins that trigger the activation of caspase-1, and effector proteins that are pro-inflammatory [21,22]. These biochemical mechanisms develop in an attempt to utilize various nutrients present in cardiomyocytes such as vitamin C, E and beta carotene as well as possibly flavanols, which are potential antioxidants for the protection against enormous oxidative stress developed in HF patients [21,22]. The increase in homocysteine related to oxidative stress is antagonized by vitamins B6, B12 and folic acid. L-carnitine, coenzyme Q_10_, cysteine, taurine, magnesium and potassium may also decline due to increased requirements during oxidative stress, which may hasten morbidity and mortality in patients with HF [21,22]. Many clinical practice guidelines support a low-sodium diet and the restriction of fluids among patients with HF, and research findings indicate that a low-sodium diet may have adverse effects on myocardial metabolism, leading to arrhythmias [63]. Therefore, there is an unmet need to determine if a Mediterranean type of foods or Indo-Mediterranean-style foods rich in vegetables, whole grains, fruits, nuts, olive oil, and spices that are rich in all the micronutrients may be protective against CHF [32,33].

In a cohort study, 1140 hospitalizations for HF were made during a mean of 13 years. After multivariable adjustment (energy intake, demographics, lifestyle factors, prevalent cardiovascular disease, diabetes, hypertension), HF risk was higher with a greater intake of eggs (1.23) and high-fat dairy (1.08) and HF risk was lower with greater whole-grain intake (0.93) [32]. These associations remained significant independent of intakes of the five other food categories, which were not associated with HF. It is possible that consumption of whole-grain was positively associated with a decreased risk of HF, whereas egg consumption and dairy products with high-fat showed higher HF risk [32]. The Physicians’ Health Study (1982–2008) studied 21,120 apparently healthy men (mean age 54.6 years) for approximately 26 years [33]. The results showed a positive and graded relation between intake of red meat and risk of HF (hazard ratio (95% CI) of 1.0, 1.02, 1.08, 1.17, and 1.24 from the lower most to the highest quintile of intake of red meat, respectively (*p* for trend 0.007)) [33]. This observation of association was for HF with (p for trend 0. 035) and without (p for trend 0.038) history of myocardial infarction [10,33]. In another cohort study involving 15,362 participants, the frequency of intake of fried food was examined by a food frequency method [64]. After a follow-up of 9.6 ± 2.4 years, there were 632 new cases of HF. Fried food intake of <1 per week: HRs (95% CI) for HF were 1.24, 1.28, and 2.03 for fried food intake of 1 to 3/week, 4 to 6/week, and 7+/week, respectively, (*p* linear trend, 0.0002) [64]. The findings were similar for consumption of fried foods at home or away from home and among people with greater scores of diet or HF. The findings indicate a positive association of frequency of fried food consumption with incident HF [64].

Another study [65] included 16,068 subjects, aged mean 64.0 ± 9.1 years, 58.7% females, 33.6% nonwhite, 34.0% living in stroke area. After a follow-up of median of 8.7 years, 363 subjects had hospitalizations for incident HF [65]. The highest adherence to the Southern dietary pattern—refined foods, fried foods, red meat and processed meat, and sweetened foods—was correlated with a 72% greater risk of HF after adjusting for various confounders; hazard ratio: 1.72; *p* = 0.005 [65]. However, plant-based dietary intakes with the topmost quartile of adherence, compared with the lowest quartile, were associated with a 41% decline in HF risk; hazard ratio: 0.59; *p* = 0.004. No associations were observed with the other three dietary patterns. It is possible that adherence to a plant-based dietary pattern was inversely associated with incident HF risk, whereas the Southern dietary pattern was positively associated with incident HF risk [47,65].

Another analysis [66] comprised 2441 males aged 42 to 60 years at entry to study, in the Kuopio Ischemic Heart Disease Risk Factor Study. The risk of HF was estimated according to protein consumption using Cox proportional hazard ratios. After 22.2 years of follow-up, there were 334 cases of incident HF. The consumption of greater total protein showed a trend toward higher HF risk (multivariable-adjusted hazard ratio in the highest versus lowest quartile = 1.33; *p*-trend = 0.05) [66]. There was a significant association between specific protein types with risk of incident HF. However, consistent with this overall result, other associations did not reach statistical significance. The ratio of hazard in the lowest versus highest quartile was 1.43; *p*-trend = 0.07 for total animal protein and 1.17; *p*-trend = 0.35 for total plant protein. It is possible that greater intake of protein was marginally associated with higher HF risk.

A meta-analysis involving 39 studies, with 2 million subjects, included 85,053 cases with CAD, 25,103 with stroke, 7536 with HF, and 147,124 with CVD in the assessment [67]. The analysis from 14 studies revealed that consumption of up to six eggs in a week had inverse association with risk of CVDs, as compared to no egg intake (for four eggs per week, Summary RR(SRR) = 0.95); a reduced risk of incident CVD was found for intake of up to one egg per day (SRR = 0.94). For incidence and mortality of CAD, the analysis out of 16 studies revealed a reduced risk up to two eggs in a week ((SRR = 0.96). Interestingly, the analysis for HF risk, from four studies revealed that consumption of one egg daily was associated with greater risk raising for higher intakes compared to no consumption (for one egg daily, SRR = 1.15) [67]. Using GRADE criteria for strength, it was rated low for all outcomes. No conclusive evidence was observed on the role of egg in increasing risk of CVD. Higher quality studies are urgently warranted to find stronger evidence for a possible protection from CVD associated with egg intake compared to not eating. It seems that future research is necessary to demonstrate the role of egg intake for increased risk of HF. There is no mention of taking designer egg containing w-3 fatty acids and tea flavonoids being protective against CVDs, including HF.

## 10. Protective Dietary Patterns in the Prevention of Heart Failure

In the USA, as well as in other Western countries, dietary patterns of patients with HF reveal a generally poor Western-type diet that may have a negative impact and a Mediterranean-style diet that may have a beneficial effect on pathophysiology and progression of CVDs and HF [46,52,53]. The Dietary Approaches to Stop Hypertension (DASH) diet, is also a Mediterranean-style diet. Epidemiological studies indicated that the incidence and risk of HF is significantly lower in patients who continue to follow this diet, which emphasizes that lower intake of saturated fat and high consumption of PUFA, complex carbohydrates, fruits, spices and vegetables [51,52,53,54,55,68,69] is beneficial. In dietary trials in patients with CVDs, these diets have been found to have beneficial effects on HF [70,71].

There is evidence that alterations in nutritional status, such as deficiency of fatty acids and amino acids, may predispose individuals to oxidative stress, leading to an increased risk of HF [55,56,57,58]. The association between glutamate and glutamine in relation to cardiometabolic disorders has been evaluated, in the development of atrial fibrillation (AF) and HF among 509 incident cases of AF, 326 with HF and 618 control subjects [72]. After a follow-up of 10 years, glutamate was associated with a 29% greater risk of HF and glutamine-to-glutamate ratio with a 20% reduced risk. Interestingly, glutamine-to-glutamate ratio was also inversely associated with risk of HF (OR per 1-SD increment: 0.80, when comparing extreme quartiles). Increase in glutamate concentrations were found to have a worse risk of cardiometabolic state, whereas a greater glutamine-to-glutamate ratio showed with an improvement in the risk profile. It is possible that high plasma glutamate levels possibly due to changes in the glutamate-glutamine cycle may contribute to the development of HF in subjects at greater risk of CVD [72]. There are no large-scale randomized, controlled intervention trials in patients with HF, to demonstrate the role of the Mediterranean-style diets or the HEART diets in the management of HF. There is also further evidence from experimental and clinical studies to elucidate the mechanisms of cardiac hypertrophy and HF [72,73,74,75,76,77,78,79,80,81,82,83,84,85]. In a previous study, metabolic products of the intestinal microbiom have been found to predispose individuals to atherosclerosis, which is a risk factor of HF [81].There is growing evidence on the role of egg on risk of CVDs, which may be erroneous [83]. Cardiac imaging via speckle tracking echocardiography and MRI may be useful in determining the role of nutritional factors and biomarkers in the pathogenesis of HF.

## 11. Conclusions

It is possible that increased intake of certain nutrients and foods, such as saturated fat, trans fat, sugar, red meat and preserved meat, has adverse effects, whereas glutamine and MUFA, PUFA, flavonoids and polyphenolics, omega-3 fatty acids, and other phytochemicals appear to have beneficial effects. Increased intake of unhealthy foods and nutrients may result in oxidative damage to proteins, enzymes, fatty acids and DNA in the biochemical composition, molecular structure, and function of different subcellular organelles of the heart, with oxidative myocardial dysfunction, pathological subcellular remodeling causing HF. The subcellular remodeling may be physiological or pathological and may be intimately involved in the transition of cardiac hypertrophy to HF depending on the optimal availability of useful or unhealthy food and nutrients, antioxidants, fatty acids and amino acids in the tissues. It is proposed that adherence or non-adherence to the HEART diet may allow us to classify HF into six stages, based on STE findings showing dysfunctional twist and sub-endocardial dysfunction. These findings may be useful in the early diagnosis of HF in its early stages of A, B and C, which may be reversed via increased adherence to the HEART diet. It is possible that apart from hypertrophy of cardiomyocytes, new generation of cardiomyocytes predominates over the death of these cells and contributes significantly to organ growth during adulthood and in physiological remodeling. The growth of cardiac cells may be under the influence of protective nutrients such as peptides, fatty acids and flavonoids. Clinical and preclinical studies indicate that lower intake of n-3 PUFA (approximately 0.4 to 2% of energy intake) may change the composition of cardiac cell membrane fatty acids of phospholipid and reduce the onset of new HF; according to such studies, it also delays the progression of existing HF. This beneficial effect of PUFA, in particular, in conjunction with MUFA, flavonoids and other nutrients, may be associated with a decrease in oxidative dysfunction and inflammation as well as in improved resistance to mitochondrial permeability transition and prevention of HFrEF. There is an unmet need to conduct large clinical trials with an appropriately optimal HEART diet in established HF or in the primary prevention of HF to establish its role in the management of CHF.

## Figures and Tables

**Table 1:** Clinical and echocardiographic features of six stages of heart failure with oxidative dysfunction.

Stages (HT on HF)	Manifestations	2D Echocardio Graphic	2D Speckle Tracking Echo	3D Speckle Tracking Echo
Stage A	Mild to moderate Oxidative dysfunction, neuro-humoral dysfunction begins.	Increasing filling pressure with abnormal relaxation	Dysfunctional untwist rate LA strain reduced.	Dysfunctional Untwist Rate, LA strain reduced.
Stage B	Moderate oxidative dysfunction, hyper-rotation. Sub-endocardial dysfunction.	Dysfunction of systole	Dysfunctional untwist rate and Increased diastolic pressure. LA strain decreased	Dysfunctional untwist rate and Increased diastolic pressure. LA strain decreased
Stage C, PHF	Asymptomatic Physio-Pathological remodeling+	EF% normal > 53%	Normal GLS −20–−23% ≥−27.0% area strain	Normal GLS −17–21% Normal AS −31–−36 %
Stage D, PHF and HFpEF	Pathological remodeling disease without symptoms of HF but elevated Natriuretic peptide, dyspnea on exertion	EF% ≥ 50% Systolic LV dysfunction.	EF% 40–49% Impaired GLS–16–20% Impaired GCS, GRS Impaired early diastolic SR, right ventricular LS, and global RV longitudinal SR	Impaired GLS –16–20% Impaired AS −27–31%
Stage E, HFmrEF	Structural heart disease with symptoms of HF	EF% 40–49% Grade 1, diastolic dysfunction	Reduced GLS−12–16% Reduced GCS, GRS, Treat with ACE,ARB.ARNI	GLS ≤ −16% AS ≤ −27 %
Stage F, HFrEF	Refractory class III HF	EF% < 40%	All above GLS < −12% Treated with ARNI	GLS < −13% AS < −27%

GLS = global longitudinal strain, GCS = global circumferential strain, GRS = global radial strain, LV = left ventricle, SR = strain rate, ARNI = angiotensin receptor neprilysin inhibitor, mr EF= mild reduction in Ejection fraction (EF), r = reduction in EF, LA = left atrial (modified from the following references: [3,12]).

**Table 2:** Antioxidant defences and antioxidants available in the HEART diet.

Indogenous Antioxidants	Exogenous Antioxidants from HEART Diet
Enzymes	Vitamins
Superoxide dismutase (SOD)	Vitamin C, ascorbic acid, ascorbate
Glutathion peroxidase (GPS)	Vitaminss, E, tocopherol, tocotrienol
Glutathion reductase	Vitamin A, vitamin D
Glutathion-S-transferase	Polyphenolics and favonoids
Paraoxanase	Quercitin, resveratrol
Thioredoxin reductase	Catechins; Flavonols, Flavanols
Heme- oxygenase	Curcumin
Aldehyde dehydrogenase	Anthrocyanins
8-Oxyguanine glycoselase	Phenolic acid
Catalase (Iron dependent)	Isoflavons/Genestein
Non-enzyme antioxidant	Carotinoids
Bilirubin	Alpha-carotine, beta-carotine
Coenzyme Q10	Zeaxanthin
L-carnitine	Lutein
Alpha-lipoic acid	Lycopine
Melatonin	Beta-cryptixanthin
Uric acid, cholesterol	Minerals
Metal binding proteins	Magnesium
Metallothioneine	Selinium, cromium
Lactoferrin	Zinc, copper
Transferrin	Fiber in the diet; oligosaccharides, polysaccharides
Ferritin	Fatty acids; Omega-3 and Monounsaturated
Ceruloplasmin (Cu dependent)	Amino acids; L-theanine, arginine, L-tryptophan
